# A Comparative Analysis of Objective Structured Clinical Examinations (OSCEs) and Traditional Written Exams in Medical Education for Assessing Clinical Competence

**DOI:** 10.7759/cureus.102867

**Published:** 2026-02-03

**Authors:** Purushotham G, G.S.R. Hareesh, Lakshmi M Jasti, Haasa Chandrika

**Affiliations:** 1 Department of General Surgery, Government Medical College, Ongole, IND

**Keywords:** comparitive study, government college india, long-case written assessment, osce, traditional clinical case examination

## Abstract

Objective

This study aimed to compare the effectiveness of traditional written examinations and Objective Structured Clinical Examinations (OSCEs) in assessing clinical competence among medical students. Additionally, it sought to explore the practical challenges and opportunities of integrating OSCEs into undergraduate curricula, evaluate the validity and reliability of both assessment methods, and propose a standardized framework to support consistent and objective evaluation.

Methodology

A total of 120 phase III part II Bachelor of Medicine, Bachelor of Surgery (MBBS) students were enrolled and randomly divided into two groups. Group A was assessed using traditional long-case written examinations, while group B underwent OSCEs. Feedback from students and examiners was collected using a pre-designed questionnaire evaluating clarity, fairness, anxiety levels, and the ability to demonstrate clinical skills effectively.

Results

The mean total score in group A was 62.02 ± 5.82, whereas group B achieved a significantly higher mean score of 75.63 ± 5.28 (p=0.0001), indicating superior performance in the OSCE group. Examiners reported that the OSCE was feasible and sustainable, and that it provided a more objective assessment of clinical competence.

Conclusions

The OSCE meets nearly all criteria for an ideal clinical competency assessment. Its structured, objective, and reliable design makes it highly suitable for evaluating clinical skills. Given its demonstrated effectiveness and practicality, the OSCE can be adopted as a standard evaluation method across diverse clinical disciplines.

## Introduction

Assessing clinical competency is a cornerstone of medical education, ensuring that future physicians possess the necessary skills and can apply them effectively in real-world clinical settings [1,2]. Evaluating the ability of medical students to translate theoretical knowledge into clinical practice has become a central focus of educational reform, particularly in an era emphasizing patient safety, communication, and professionalism [3]. The choice of assessment method is therefore critical, as evaluation profoundly influences learning behavior and plays a key role in shaping skilled, confident, and compassionate practitioners [4].

Historically, medical schools have relied on oral examinations, long and short case presentations, and conventional written tests to assess clinical competence [5]. Although these methods assess theoretical knowledge and specific reasoning skills, they often lack objectivity and standardization, leading to considerable variability in examiner judgment and differences in case complexity [6]. Moreover, traditional assessments primarily address the lower levels of Miller's Pyramid-"know" and "know how"-providing limited insight into higher levels such as "show how" and "do", which reflect actual clinical performance [7].

To overcome these limitations, Harden introduced the Objective Structured Clinical Examination (OSCE) in the 1970s as a systematic and objective approach to assessing clinical and communication skills [8]. In an OSCE, students rotate through timed stations that simulate authentic clinical scenarios, with each station designed to assess specific competencies, including patient interviewing, physical examination, diagnostic reasoning, and management planning [9]. The use of standardized patients and structured checklists enhances objectivity, fairness, and reliability, establishing OSCEs as a widely accepted tool for competency-based assessment.

Currently, OSCEs are regarded as the gold standard for evaluating clinical competence and are extensively implemented in undergraduate and postgraduate medical programs across the United States, Canada, Australia, and Europe [9]. Despite these advantages, OSCEs are resource-intensive, requiring trained examiners, simulated patients, and substantial logistical support [10]. Furthermore, existing literature reports mixed findings regarding their correlation with traditional written and oral assessments [10-12].

These considerations highlight the need to determine whether OSCEs truly provide a superior measure of clinical competency and how effectively they can complement conventional assessment methods. In the context of the global shift toward competency-based medical education, a critical appraisal of the strengths, limitations, and applicability of different evaluation strategies is essential.

The present study aims to compare the effectiveness of traditional written examinations and OSCEs in assessing clinical competence among medical students. Additionally, it seeks to examine the practical challenges and opportunities associated with integrating OSCEs into undergraduate curricula, evaluate the validity and reliability of both assessment methods, and propose a standardized framework to promote consistent and objective evaluation.

## Materials and methods

Study design and population

This comparative study was conducted in the Department of General Surgery at Government General Hospital and Government Medical College, Ongole, Andhra Pradesh, over six months from May to October 2025. The study population consisted of phase III part II Bachelor of Medicine, Bachelor of Surgery (MBBS) students undergoing clinical rotations in general surgery. Assessments were performed after their surgical postings to compare the effectiveness of traditional long-case written assessments and OSCEs in measuring clinical competence. The study was conducted after obtaining approval from the Institutional Ethics Committee under reference number IEC/GMC-OGL/248/2025.

Sample size

A total of 120 phase III part II MBBS students participated in the study. Each participant was assigned a unique identification number (1-120) and randomly allocated to group A or group B using simple random sampling.

Inclusion and exclusion criteria

All phase III part II MBBS students who were posted in the Department of General Surgery during the study period and had completed the required clinical rotation were eligible for inclusion. Students who were absent during the assessment period or did not complete the assigned evaluation were excluded. Written informed consent was obtained from all participants prior to enrollment.

Data collection

Group A was assessed using the conventional long-case written examination method. Each student was assigned a real patient from the surgical wards and was required to obtain a detailed clinical history, perform a comprehensive physical examination, formulate a diagnosis with appropriate differential diagnoses, and develop a suitable management plan. Performance was evaluated independently by four examiners at a single assessment site using predefined scoring criteria to ensure objectivity and consistency.

Group B was assessed using the OSCE format. Each student rotated through four stations, including one rest station. The active stations evaluated key clinical competencies, including history taking, physical examination, management planning, diagnostic interpretation, and communication skills.

The history-taking station employed a standardized patient to assess the student's ability to elicit relevant clinical information. The physical examination and management planning stations involved real patients to evaluate examination techniques and treatment-planning skills. The diagnostic interpretation station assessed the ability to analyze clinical data, including laboratory investigations, radiographic findings, and biopsy reports. The communication skills station evaluated the student's proficiency in explaining surgical procedures, including associated risks and benefits, to patients.

Each OSCE station was supervised by a qualified examiner who used a structured checklist to evaluate performance. Scores from individual stations were systematically recorded, and OSCE outcomes were subsequently compared with those of the traditional long-case written examination. Upon completing the assessments, students received their results and provided structured feedback through a predesigned questionnaire about their experiences with both evaluation methods.

Statistical analysis

Data were entered into Microsoft Excel (Microsoft, Redmond, Washington) and analyzed using the Statistical Package for the Social Sciences (SPSS), version 25.0 (IBM Inc., Armonk, New York). Descriptive statistics, including means, standard deviations, and percentages, were used to summarize quantitative variables. Inferential analysis was performed using appropriate statistical tests, such as the paired t-test, to compare outcomes between the two groups. A p-value of less than 0.05 was considered statistically significant.

## Results

A total of 120 participants were included, with 60 students in each group. The mean age of students in group A (traditional exam) was 20.95 ± 0.85 years, while in group B (OSCE) it was 21.05 ± 0.77 years. Group A comprised 32 (53%) males and 28 (47%) females, whereas group B included 31 (52%) males and 29 (48%) females (Table 1).

**Table 1:** Demographic characteristics of participants OSCE - Objective Structured Clinical Examination

Variable		Group A Traditional exam (n=60)	Group B OSCE (n=60)
Age		20.95 + 0.85	21.05 + 0.77
Gender	Male	32 (53%)	31 (47%)
	Female	28 (52%)	29 (48%)

All participants in both groups had prior experience with traditional written examinations, but none had previously encountered the OSCE format (Table 2).

**Table 2:** Previous exposure of participants OSCE - Objective Structured Clinical Examination

Variable	Group A	Group B
Traditional written exam	Yes	Yes
OSCE	No	No

The overall perception score was significantly higher for OSCE (6.63 ± 0.96) compared to the traditional examination (6.27 ± 0.95), indicating a more favorable student perception of the OSCE format (t=2.21, p=0.03) (Table 3).

**Table 3:** Comparison of scores between group A - traditional long-case evaluation and group B - OSCE assessment exams Scores between the traditional written examination and OSCE were compared using a paired t-test. A p-value <0.005 was considered statistically significant. ** shows significant p-value OSCE - Objective Structured Clinical Examination

Assessment method	Mean score	Max score	Min score	Statistical test value	P-value
Traditional written exam	62.02 + 5.82	71	40	14.26	0.0001**
OSCE	75.63 + 5.28	88	65		

Analysis of performance across different OSCE stations revealed the highest mean scores in physical examination and case presentation (mean=8), followed by history taking and communication skills (mean=7 each). Maximum scores across stations ranged from 7-9, while minimum scores were consistently 6 (Table 4).

**Table 4:** Student performance in different OSCE stations OSCE - Objective Structured Clinical Examination

OSCE station	Mean score	Max score	Min score
History taking	7	8	6
Physical examination	8	7	6
Case presentation	8	9	6
Communication skills	7	8	6

The mean score in the traditional written examination was 62.02 ± 5.82, with a range of 40-71, whereas the mean score in the OSCE was higher at 75.63 ± 5.28, ranging from 65 to 88. The difference between the two assessment methods was statistically significant (t=14.26, p=0.0001), indicating superior performance in the OSCE. Students also rated the clarity of assessment criteria higher in the OSCE (6.57 ± 1.06) than in the traditional examination (6.15 ± 1.05), with this difference reaching statistical significance (t=2.18, p=0.03). Similarly, the perceived opportunity to demonstrate skills was higher in OSCE (6.63 ± 0.99) than in the traditional examination (6.27 ± 0.97), showing a statistically significant difference (t=2.05, p=0.04). Although the mean score for fairness was higher in OSCE (6.40 ± 1.39) than in the traditional examination (5.95 ± 1.41), this difference was not statistically significant (t=1.77, p=0.08). Anxiety levels before the examination were slightly higher in the OSCE (7.10 ± 0.89) than in the traditional format (6.90 ± 1.02), but the difference was not statistically significant (t=1.13, p=0.26) (Table 5).

**Table 5:** Feedback from students on assessment methods Scores between the traditional written examination and OSCE were compared using a paired t-test. A p-value <0.005 was considered statistically significant. * shows significant p-value OSCE - Objective Structured Clinical Examination

Question	Traditional exam	OSCE	Student t-test value	p-value
Clarity of assessment criteria	6.15 + 1.05	6.57 + 1.06	2.18	0.03*
Fairness in assessment	5.95 + 1.41	6.40 + 1.392	1.77	0.08*
Ability to demonstrate skills	6.27 + 0.97	6.63 + 0.99	2.05	0.04*
Anxiety levels before the exam	6.90 + 1.02	7.10 + 0.89	1.13	0.26
Overall	6.27 + 0.95	6.63 + 0.96	2.21	0.03*

## Discussion

This study compared the effectiveness of Objective Structured Clinical Examinations (OSCEs) and traditional long-case written examinations in assessing clinical competence among phase III part II MBBS students. The two groups were demographically comparable in terms of age and gender distribution, and importantly, neither group had prior exposure to the OSCE format. This minimized the influence of familiarity and ensured that observed differences in performance were attributable primarily to the assessment methods.

The results demonstrated that students assessed using OSCEs achieved significantly higher mean scores (75.63 ± 5.28) than those evaluated through the traditional long-case examination (62.02 ± 5.82), with a p-value of 0.0001. These findings suggest that OSCEs provide a more comprehensive and objective measure of clinical competence. Similar conclusions have been reported by Ponnamperuma et al. [13] and Newble [14], who noted that the structured, criterion-referenced nature of OSCEs enhances discrimination and improves score consistency across varying levels of student competence. The wider score range and greater standard deviation observed in the OSCE group further indicate a superior ability to differentiate between performance levels, consistent with findings by Wass and van der Vleuten [15].

Although the traditional long-case examination remains valuable for assessing holistic patient management, it has been widely criticized for subjectivity, examiner variability, and lack of standardization in clinical encounters. These limitations likely contributed to the lower mean scores and more clustered performance observed in this group.

Analysis of individual OSCE stations revealed consistent mean scores ranging from seven to eight across history, taking physical examination, case presentation, and communication skills. This consistency indicates that OSCEs effectively assess both technical and interpersonal competencies, supporting Harden's original assertion that OSCEs represent a rigorous and objective evaluation method [8]. Student feedback further reinforced these findings, with participants rating OSCEs higher on fairness, opportunities to demonstrate skills, and clarity of assessment criteria (p=0.03). These perceptions align with previous studies reporting that OSCEs are viewed as more equitable and transparent than traditional assessments [8,13].

In line with observations by Siddaram and Anil [16], this study found that although initial anxiety associated with OSCEs was slightly higher, it diminished with repeated exposure and did not negatively affect overall performance. Prior reviews [17,18] have emphasized the reliability and validity of OSCEs while reporting low to moderate correlations with other assessment formats. For instance, Alunno et al. [17], in an analysis of 36 studies evaluating resident competence, reported minimal correlation between OSCE and multiple-choice question (MCQ) scores despite high internal consistency and inter-rater reliability. This supports the conclusion that OSCEs assess a distinct construct encompassing clinical performance, communication skills, and applied knowledge.

Previous research has similarly reported low correlation coefficients, typically below 0.50, between standardized patient-based examinations and other assessment modalities, including clinical ratings, short-answer tests, MCQs, and board examinations [19,20]. Such weak correlations are expected, as OSCEs evaluate different dimensions of competence and are often administered at various stages of training. However, some authors have suggested that limited validity evidence may also contribute to these modest associations [19].

Two randomized studies [21,22] reported no significant differences among various OSCE formats, supporting their comparability. Nevertheless, due to the limited number of studies in this area [19-22], further research is needed to determine whether online OSCEs can adequately assess complex competencies, such as teamwork, leadership, and professionalism, compared with in-person examinations.

The rapid adoption of online OSCEs during the COVID-19 pandemic represented a significant shift in medical education. Overall satisfaction with online formats has been reported among standardized patients, instructors, and trainees [22-25], suggesting their feasibility for selected station types. However, technical challenges and the difficulty of assessing physical examination skills remotely remain significant limitations to their widespread implementation [26].

Our study adds to the growing evidence supporting the reliability, validity, and acceptability of OSCEs as practical tools for evaluating clinical competence. Although OSCEs require substantial resources and examiner training, their structured design enhances fairness, reduces bias, and enables comprehensive assessment across multiple domains of clinical skill. Continued refinement of OSCE design, faculty development, and thoughtful curricular integration will further strengthen their role in undergraduate medical education.

Limitations

This study has several limitations that should be acknowledged. It was conducted at a single institution and involved only phase III part II MBBS students from the Department of General Surgery, which may limit the generalizability of the findings to other settings, academic levels, and clinical disciplines. Although random allocation was used, a crossover design was not employed, and baseline differences in student aptitude or learning styles could have influenced performance outcomes. Additionally, none of the participants had prior exposure to OSCEs, and the novelty of this assessment format may have affected student motivation and scores. The OSCE consisted of a limited number of stations conducted during a single examination session, which may not fully capture the breadth of clinical competence. Despite the use of structured checklists, examiner-related variability cannot be completely excluded. Student feedback was based on self-reported questionnaires and is therefore subject to response bias. Finally, the study assessed short-term performance only and did not evaluate long-term retention of clinical skills or real-world clinical competence.

## Conclusions

This study demonstrates that the OSCE meets the key criteria for a practical clinical competency assessment. It is practically feasible within the institutional context while providing a balanced combination of objectivity, validity, and reliability. Further studies with larger sample sizes are warranted to confirm its consistency and generalizability. Notably, the OSCE can be successfully implemented across diverse clinical disciplines to enhance the overall quality of medical education.

## References

[1] Spanke J, Raus C, Haase A (2019). Fairness and objectivity of a multiple scenario objective structured clinical examination. GMS J Med Educ.

[2] Fowell SL, Bligh JG (1998). Recent developments in assessing medical students. Postgrad Med J.

[3] Zayyan M (2011). Objective structured clinical examination: the assessment of choice. Oman Med J.

[4] Eldarir SA, Nagwa A, Hamid A (2013). Objective structured clinical evaluation (OSCE) versus traditional clinical students’ achievement at maternity nursing: a comparative approach. IOSR J Dent Med Sci.

[5] Khan M, Noor SM, Siraj MU (2015). Students' perceptions of OSCE in dentistry: a study from Khyber College of Dentistry, Pakistan. Adv Health Prof Educ.

[6] Onwudiegwu U (2018). OSCE: design, development and deployment. J West Afr Coll Surg.

[7] Miller GE (1990). The assessment of clinical skills/competence/performance. Acad Med.

[8] Harden RM, Stevenson M, Downie WW, Wilson GM (1975). Assessment of clinical competence using objective structured examination. Br Med J.

[9] Khan KZ, Gaunt K, Ramachandran S, Pushkar P (2013). The objective structured clinical examination (OSCE): Amee Guide No. 81. Part II: organisation & administration. Med Teach.

[10] Chang O, Holbrook AM, Lohit S, Deng J, Xu J, Lee M, Cheng A (2023). Comparability of objective structured clinical examinations (OSCEs) and written tests for assessing medical school students' competencies: a scoping review. Eval Health Prof.

[11] Bevan J, Russell B, Marshall B (2019). A new approach to OSCE preparation - PrOSCEs. BMC Med Educ.

[12] Wagner FL, Feller S, Schmitz FM, Zimmermann PG, Krings R, Guttormsen S, Huwendiek S (2022). Usability and preference of electronic vs. paper and pencil OSCE checklists by examiners and influence of checklist type on missed ratings in the Swiss Federal Licensing Exam. GMS J Med Educ.

[13] Ponnamperuma GG, Karunathilake IM, McAleer S, Davis MH (2009). The long case and its modifications: a literature review. Med Educ.

[14] Newble D (2004). Techniques for measuring clinical competence: objective structured clinical examinations. Med Educ.

[15] Wass V, van der Vleuten C (2004). The long case. Med Educ.

[16] Mondal R, Sarkar S, Nandi M, Hazra A (2012). Comparative analysis between objective structured clinical examination (OSCE) and conventional examination (CE) as a formative evaluation tool in Pediatrics in semester examination for final MBBS students. Kathmandu Univ Med J (KUMJ).

[17] Alunno A, Najm A, Sivera F, Haines C, Falzon L, Ramiro S (2020). Assessment of competences in rheumatology training: results of a systematic literature review to inform EULAR points to consider. RMD Open.

[18] Walsh M, Bailey PH, Koren I (2009). Objective structured clinical evaluation of clinical competence: an integrative review. J Adv Nurs.

[19] Turner JL, Dankoski ME (2008). Objective structured clinical exams: a critical review. Fam Med.

[20] van der Vleuten CPM, Swanson DB (1990). Assessment of clinical skills with standardized patients: state of the art. Teach Learn Med.

[21] Biolik A, Heide S, Lessig R (2018). Objective structured clinical examination "Death Certificate" station - Computer-based versus conventional exam format. J Forensic Leg Med.

[22] Oliven A, Nave R, Gilad D, Barch A (2011). Implementation of a web-based interactive virtual patient case simulation as a training and assessment tool for medical students. Stud Health Technol Inform.

[23] Farrell SE, Junkin AR, Hayden EM (2022). Assessing clinical skills via telehealth objective standardized clinical examination: feasibility, acceptability, comparability, and educational value. Telemed J E Health.

[24] Kelly R, Leung G, Lindstrom H, Wunder S, Yu JC (2022). Virtual objective structured clinical examination: experiences and performance in physical medicine and rehabilitation residency. Am J Phys Med Rehabil.

[25] Martinez L, Holley A, Brown S, Abid A (2020). Addressing the rapidly increasing need for telemedicine education for future physicians. PRiMER.

[26] Kupfer M (2025). Med school final exam plagued with technical issues after moving online due to COVID-19. CBC news. CBC News.

